# Conjunctivitis as sole symptom of COVID-19: A case report and review of literature

**DOI:** 10.1177/1120672120946287

**Published:** 2020-07-24

**Authors:** Zeynep Kayaarasi Ozturker

**Affiliations:** Department of Ophthalmology, Baskent University Istanbul Hospital, Istanbul, Turkey

**Keywords:** Conjunctivitis, COVID-19, novel coronavirus, ocular infection, SARS-CoV-2

## Abstract

**Introduction:**

Severe acute respiratory syndrome coronavirus 2 (SARS-CoV-2) is a novel virus causing an ongoing pandemic in 2020. Although the symptomatic patients infected by SARS-CoV-2 generally show respiratory distress, atypical manifestations such as conjunctivitis are also observed. A series of cases is reported in which reverse transcriptase polymerase chain reaction (RT-PCR) testing on tears had demonstrated the presence of the virus. However, the transmission of the virus through ocular fluids remains unknown.

**Case description:**

In this case report, the development of conjunctivitis is presented as the sole symptom of a new coronavirus disease 2019 (COVID-19) in an emergency health care worker. The patient’s first application was to the ophthalmology clinic due to redness, stinging, tearing, and photophobia for one day in the right eye. The patient had no symptoms of fever, cough, shortness of breath, or fatigue. Two days later, the RT-PCR test, blood analysis, and chest computed tomography (CT) were applied to the patient for being in contact with a COVID positive patient. Conjunctival swabs did not identify SARS-CoV-2 by RT-PCR. However, nasopharyngeal swab and blood test confirmed the diagnosis of COVID-19. Chest CT did not show pneumonia.

**Conclusion:**

This phenomenon shows that conjunctivitis may occur as a sole manifestation of COVID-19 which needs to be carefully evaluated by health care workers and eye care professionals during the pandemic.

## Introduction

Coronavirus Disease 2019 (COVID-19) is a highly contagious newly recognized infection that has a significant worldwide impact on mortality and economic morbidity. Initial reports established from respiratory samples revealed severe acute respiratory syndrome coronavirus 2 (SARS-CoV-2) as the causative. Generally, patients infected with SARS-CoV-2 develop respiratory illness, with the first symptoms of fever, cough, and fatigue that quickly progress to pneumonia. Several patients were observed with atypical manifestations at the onset of the illness, such as conjunctivitis, or even presented with asymptomatic infection.

SARS-CoV-2 gains entry into host cells by binding to the angiotensin-converting enzyme 2 (ACE-2) receptor, which is distributed among various tissues, including the conjunctiva.^1^ During the SARS associated coronavirus outbreak of 2003, a study indicated that healthcare workers suffered from a higher risk of SARS infection when there was unprotected eye contact with secretions.^2^ There are increasing reports suggesting that a few COVID-19 pneumonia cases began with conjunctivitis as the initial symptom following contact with confirmed patients. Detection of viral RNA by reverse transcriptase polymerase chain reaction (RT-PCR) can be useful in early detection of SARS-CoV-2 infection and taking appropriate quarantine measures. Therefore, determining whether SARS-CoV-2 is capable of transmitting through contact with conjunctiva is an important consideration that warrants for exploration.

Here, we present a case report of a health care worker diagnosed with COVID-19 who experienced conjunctivitis as the first and sole symptom of the disease. We believe it is important for healthcare practitioners who play a significant role in the battle against the pandemic, to be knowledgeable about this problem and to take necessary steps to prevent the spread of the disease.

The study followed the tenets of the Declaration of Helsinki. The patient signed written informed consent for the research use of clinical records and data included in the study.

## Case description

**Figure 1.**
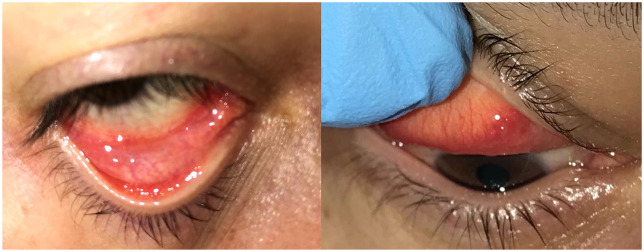
Follicular conjunctival reaction in the upper and lower fornices, serous secretion, and mild chemosis in the right eye of the patient 2 days before the diagnosis of COVID-19.

A 32-year-old otherwise healthy nurse working in the Emergency Department of Baskent University presented to the ophthalmology clinic on 8 May 2020 with one day history of redness, stinging, watery discharge, and photophobia in his right eye. The patient had no symptoms of fever, cough, shortness of breath, or general malaise. In his story, he did not declare any travel abroad in the last 14 days. The patient was diagnosed with idiopathic anterior uveitis in the right eye 2 years ago. Due to the regulations determined by the Turkish Republic Ministry of Health Department, oral and nasopharyngeal swab tests for SARS-CoV-2 were recommended for health care workers on demand. Upon the patient’s request, RT-PCR tests applied on 14 April 2020 and 24 April 2020 had negative results consequently. In his ophthalmic examination, the visual acuity was 20/20 for both eyes without correction. Intraocular pressure was 13 mmHg on the right and 14 mmHg on the left eye. Slit-lamp examination of the right eye revealed eyelid edema and serous secretion with 2+ conjunctival injection, mild chemosis, and follicular reaction in the upper and lower fornices (Figure 1). The cornea was transparent, and no sign of inflammation was detected in the anterior chamber. Fundus examination revealed vital optic disc and macula. Anterior and posterior segment examination of the left eye was normal. Physical examination did not show any tenderness or enlargement of the submandibular, preauricular, or cervical lymph nodes. The patient declared that he wore personal protective equipment during close contact with suspected COVID-19 cases in the Emergency Department, but in some occasions, he had to remove his protective eyewear during the interventions.

**Figure 2.**
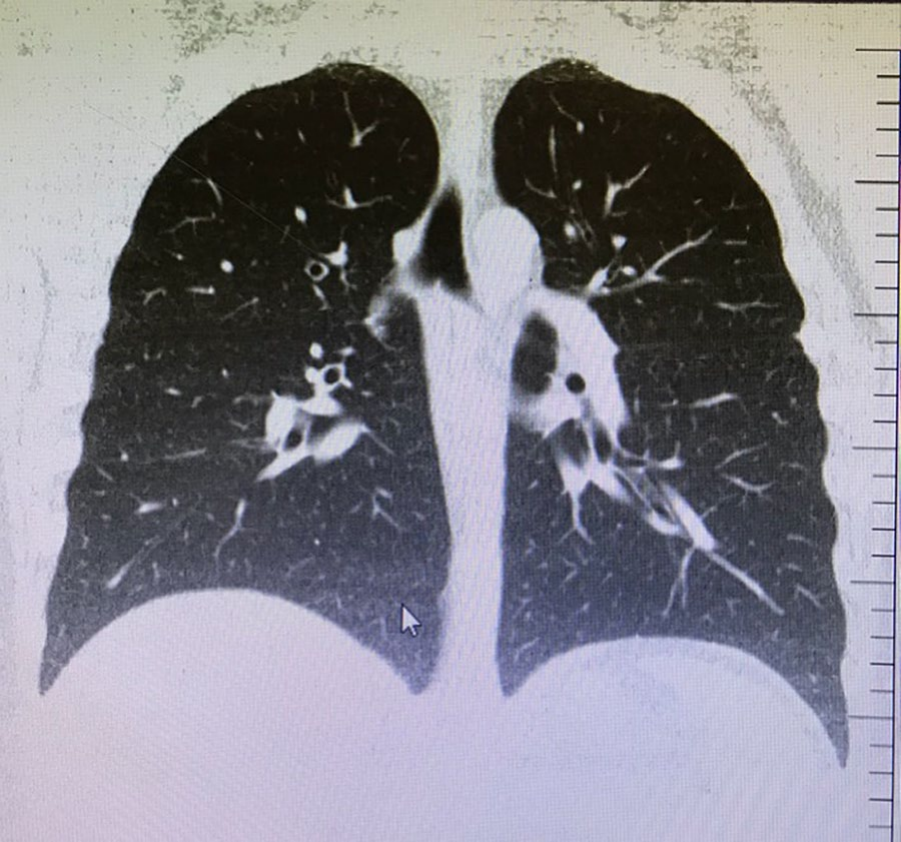
Computed tomography of the pulmonary parenchyma showing no sign of pneumonia.

Considering acute conjunctivitis, moxifloxacin eye drop QID and artificial tears without preservative QID were prescribed for 7 days. On 10 May 2020, his mother, whom he lives together, developed a cough and fatigue, and RT-PCR test for nasopharyngeal swab was declared positive for COVID-19. Given his direct contact with his mother and occupational exposure, nasopharyngeal RT-PCR test was applied to him on the same day, and a positive result was reported for SARS-CoV-2. Nasal swabs for Influenza a and b virus antigens were negative. His chest computed tomography and chest X-ray showed no signs of pneumonia (Figure 2). The routine blood examination showed high levels of glucose (114 mg/dL), C-reactive protein (13.00 mg/L), AST (44 U/L), ALT (108 U/L), LDH (251 U/L), and monocytes% (14.5%). He was started on taking systemic hydroxychloroquine and azithromycin for 5 days and instructed to self-quarantine until the complete resolution of the infection. On day 5 after diagnosis, the patient consulted his ophthalmologist by telemedicine service due to the worsening of his eye symptoms. Because of the infectious nature of COVID-19, quarantine protocols prevented access to the hospital during the active phase of the disease. Owing to the persistence of ocular complaints, the tear, and conjunctival swab samples were acquired by home visit. The samples were collected by conjunctival swab technique under the patient’s approval. Upper and lower eyelids of both eyes were everted, and two separate samples were obtained by sweeping both fornices with sterile cotton swab without topical anesthesia. The swabs were inserted into a viral transport medium in ice before being tested for SARS-CoV-2. The patient had negative RT-PCR results for eye samples. Routine adenovirus tests also yielded a negative result.

## Conclusion

Ocular diseases caused by coronaviruses are relatively rare compared to adenovirus and influenza viruses.^3^ Although it is known that the main route of transmission of the SARS-CoV-2 is through the respiratory tract, several studies have raised concerns due to infection in the unprotected eyes (Table 1). So far, it has not been clarified whether ocular secretions are contagious.

**Table 1.** Demographics and clinical data of COVID-19 patients with conjunctivitis.

Studies	Country	F/M	Age (years)	Conjunctivitis as initial or corresponding symptom	Time and result of cPCR after COVID diagnosis (days)
*Case reports*					
Zhang et al.^4^	China	F	29	Corresponding	6 (+)
Chen et al.^5^	China	M	30	Corresponding	14 (+), 17 (+), 19 (−)
Cheema et al.^6^	Canada	F	29	Initial	5 (+)
Hu et al.^7^	China	NA	70	Corresponding	25 (+), 29 (+), 31 (+), 34 (+), 36 (+), 39 (−)
Colavita et al.^8^	Italy	F	65	Initial	3 (+), 9 (+), 13 (+), 16 (+), 21 (−)
Khavandi et al.^9^	Iran	M	65	Initial	NA
Salducci and *La Torre*^10^	Diamond Princess	M	72	Initial	NA
Navel et al.^11^	France	M	63	None	20 (−)
Daruich et al.^12^	Argentina	M	27	Initial	NA
Wu et al.^13^	China	M	2	Initial	NA
Ya et al.^14^	China	1/2	Range: 16–67	2 initial 1 corresponding	NA
Scalinci and *Battagliola*^15^	Italy	1/4	Range: 41–65	All initial	NA
*Cohort studies*					
Xia et al.^16^	China	9/21	Mean 54.5 (SD: 14.1)	1/30 initial 29/30 none	7.3 ± 3.8 [1/30 (+)]
Chen et al.^17^	China	271/263	Median 40–50	3/534 initial 25/534 corresponding	NA
Seah and *Agrawal*^18^	Singapore	6/11	Median 37 Range: 20–75	1/17 corresponding	3–20 [all patients (−)]
Hong et al.^19^	China	25/31	Mean 48.0 (SD: 12.1)	6/56 initial 9/56 corresponding	7.3 ± 3.8 [1/56 (+)]
Wu et al.^20^	China	13/25	Mean 65.8 (SD: 16.6)	1/38 initial 11/38 corresponding	NA [2/28 (+)]
Zhou et al.^21^	China	68/53	Median 48 Range: 22–89	8/121 corresponding	NA [3/121 (+)]

F: female; M: male; cPCR: conjunctival swab polymerase chain reaction; SD: standart deviation; NA: non-applicable.

Based on current literature, SARS-CoV-2 can be detected in the swab samples taken from the conjunctiva by the RT-PCR method. In a study of 30 COVID positive patients, conjunctivitis developed in one patient (3.3%), which was the only symptom during the disease.^16^ Conjunctival specimen taken at days 3 and 5 gave positive results for viral RNA in the same patient, whereas all other patients had negative results. Conjunctival sac secretion of the patient was also tested for Herpes simplex virus, adenovirus, and other common viruses of conjunctivitis. However, the results were all negative indicating that the viral conjunctivitis of the patient might be related to SARS-CoV-2. Another study from the Hubei province demonstrated conjunctivitis-like ocular findings in a much larger proportion of cases (31.6%).^20^ However, conjunctival PCR was found to be positive only in two cases (5.3%). Blood test parameters, including white blood cell, neutrophil, procalcitonin C, C-reactive protein, and lactate dehydrogenase levels, have also been reported to be higher in patients with conjunctivitis. In a similar study, the proportion of positive results for conjunctival SARS-CoV-2 was 2.5% which was significantly lower than the nasopharyngeal SARS-CoV-2 rate.^21^ However, it was suggested that a single time point conjunctival sampling may lead to low rate of virus detection in ocular samples.

One study showed SARS-CoV-2 RNA in conjunctival swabs of a patient up to 21 days from the start of symptoms, a few more days after the virus became undetectable in nasal swabs. Five days later, the virus was undetectable in the conjunctival swab and was found positive again during the day 27, indicating continuous conjunctival replication of the virus.^8^ RNA sample was inoculated in Vero E-6 cell culture and cytopathic effect was observed postinoculum. In contradiction, a further study suggested that the risk of SARS-CoV-2 transmission through tears was low. In their series of 17 patients, only one patient had conjunctival injection and chemosis during the stay in the hospital. Conjunctival RT-PCR tests applied on day 3 and day 20 were negative for all patients. In this study, the virus was incubated in cell culture, but no cytopathic effect was determined.^18^

In a separate case report, a 70-year-old man admitted to hospital with fever, cough, and fatigue symptoms. His left eye was diagnosed with nasolacrimal duct stenosis. Interestingly, the sequential conjunctival RT-PCR results remained positive until about 2 weeks after the nasopharyngeal RT-PCR results came negative. In this clinically cured COVID-19 patient, the conjunctival swab of the left eye was still positive, which suggests that the obstruction of lacrimal drainage system may decrease the virus clear through the eye and provide its shedding persistently.^7^

Five studies have described biomicroscopic findings of COVID-19 patients with conjunctivitis.^4–6,9,10^ Ocular symptoms have emerged as the first symptom in all but one of these cases. Ocular complaints have been reported within a spectrum of red eye, watery discharge, photophobia, foreign body sensation, and eyelid edema. Remarkable details in the slit-lamp examination were serous secretion, follicular reaction in the upper and lower eyelid conjunctiva, chemosis, keratoconjunctivitis, and pseudomembranous inflammation. In four of these cases, SARS-CoV-2 viral RNA was detected in the swab samples taken from the conjunctiva.^4–6,9^

Conjunctivitis remained the only sign and symptom of four active COVID-19 cases with history of travel to Lombardy region in Italy.^15^ None had fever, general malaise or respiratory symptoms, consistent with our case. The authors emphasize the importance of ocular symptoms which can be overlooked if the patients do not show typical systemic symptoms of the disease.

Studies have shown that SARS-CoV-2 needs the ACE-2 receptors for cell invasion.^1^ The ACE-2 receptors are found not only in human type 2 alveolar epithelial cells but also in the cornea and conjunctiva.^22^ This suggests that ocular surface tissue may be a potential target tissue for SARS-CoV-2. Whether ocular contact with SARS-CoV-2 causes COVID-19 disease is unclear. However, according to one proposed theory, when the ocular surface comes into contact with SARS-CoV-2, virus particles can cause infection by draining the respiratory tract through the nasolacrimal canal.^18^ Therefore, it is recommended to use protective glasses or shields.

It is not yet known whether SARS-CoV-2 causes a more severe eye disease beyond keratoconjunctivitis. However, serious eye problems may not be adequately assessed in patients with high virus loads who are often treated in intensive care units for vital reasons. One report demonstrated severe ocular symptoms including tarsal hemorrhage, petechia and pseudomembranes in a 63-years-old patient who required intensive care surveillance and ventilatory support for COVID-19. Collected swabs did not identify any bacterial or viral etiology in conjunctival secretions and tears.^11^

Although conjunctivitis symptoms were generally found in adult patients, there has been one report on infected children. A 2-years-old asymptomatic boy was detected through community screening with positive results of oropharyngeal SARS-CoV-2 RNA. He developed conjunctivitis and eyelid dermatitis on day 7, which gradually disappeared in 5 days. As conjunctival swab was not taken, it is not clear whether a virus or secondary bacterial infection caused ocular symptoms.^13^

Telemedicine services may help in the diagnosis of patients with conjunctivitis amid COVID-19 lock-down. One study reported a 27-years-old male patient who used telemedicine due to redness and foreign body sensation in his left eye. Examination revealed lid edema and conjunctival hyperemia which required treatment. After 12 h, he admitted to the hospital because of added complaints of fever, cough, and dyspnea.^12^ In two other studies, ocular findings were evaluated retrospectively by making phone calls with the patients, and it was concluded that ocular symptoms were common in COVID-19 patients which might have clinical diagnostic significance.^17,19^

Recently, positive viral nucleic acid test results in conjunctival swab samples from confirmed COVID-19 patients with conjunctivitis provide objective evidence for SARS-CoV-2 ocular surface infection.

However, there are also patients with positive conjunctival RT-PCR results without any sign of conjunctivitis.^14^ Therefore, the relationship between ocular surface infection and COVID-19 and whether the disease can be transmitted through the ocular surface needs further research.

In conclusion conjunctivitis may appear as the only sign and symptom of COVID-19, and these patients may not have fever, fatigue, or respiratory symptom that may cause suspicion. The patients are generally those who report contact with COVID positive patients and therefore undergo nasopharyngeal RT-PCR test. According to a recent report, one-third of eye care professionals involved in the diagnosis and treatment of the patients during the pandemic accidentally acquired COVID-19 and demonstrated severe disease including death.^23^ Thus, all physicians and ophthalmologists should be cautious when addressing a patient with conjunctivitis and adopt proper steps for the possible ocular transmission of SARS-CoV-2 until a vaccine is available. If patients have no symptoms other than conjunctivitis, the RT-PCR test for nasopharyngeal or conjunctival swabs may help in early diagnosis of the disease.
